# Therapeutic Potentials of Secoiridoids from the Fruits of *Ligustrum lucidum* Aiton against Inflammation-Related Skin Diseases

**DOI:** 10.3390/ph15080932

**Published:** 2022-07-27

**Authors:** Sang Won Yeon, Su Ryeon Choi, Qing Liu, Yang Hee Jo, Da Hee Choi, Mi Ran Kim, Se Hwan Ryu, Solip Lee, Bang Yeon Hwang, Hyung Seo Hwang, Mi Kyeong Lee

**Affiliations:** 1 College of Pharmacy, Chungbuk National University, Cheongju 28160, Korea; sangwon1311@naver.com (S.W.Y.); qow0125@naver.com (Y.H.J.); alfm0188@naver.com (S.H.R.); dudaos000@hanmail.net (S.L.); byhwang@chungbuk.ac.kr (B.Y.H.); 2 School of Cosmetic Science and Beauty Biotechnology, Semyung University, Jecheon 27136, Korea; tryu0925@naver.com (S.R.C.); chlekgml930@gmail.com (D.H.C.); alfks4050@naver.com (M.R.K.); 3 Food and Pharmacy College, Xuchang University, Xuchang 461000, China; liuqing7115@hotmail.com

**Keywords:** *Ligustrum lucidum*, secoligulene, anti-inflammation, iridoid, psoriasis model

## Abstract

*Ligustrum lucidum* Aiton is a flowering plant of the Oleaceae family, and its fruits have been traditionally used for skin nourishment and the treatment of skin diseases. However, the anti-inflammatory constituents for skin disease are not well-characterized. Phytochemical investigation of *L. lucidum* fruits resulted in the isolation of a new secoiridoid, secoligulene (**1**), together with (*E*)-3-(1-oxobut-2-en-2-yl)pentanedioic acid (**2**) and *trans*-(*E*)-3-(1-oxobut-2-en-2-yl)glutaric acid (**3**). Secoligulene (**1**) displayed the potent inhibitory effect on NO production with an IC_50_ value of 12.0 μg/mL. Secoligulene (**1**) also downregulated mRNA transcriptional levels of pro-inflammatory cytokines such as IL-1 α, IL-1β, IL-6 and COX-2 in LPS-stimulated RAW264.7 cells. Further investigation showed that secoligulene (**1**) inhibited the phosphorylation of IκB and JNK activated by LPS. In addition, secoligulene (**1**) downregulated the expression of chemokines such as CXCL8 and CCL20 in the TNF-α/IL-17/IFN-γ induced HaCaT psoriasis model. Taken together, these findings support the beneficial effects of *L. lucidum* and its constituents on inflammation-related skin diseases and can be further developed as therapeutic treatments for related diseases.

## 1. Introduction

*Ligustrum lucidum* Aiton is a flowering plant of the Oleaceae family that is widely distributed all over the world in Asia, Europe and America. It has been used for ornamental purposes and as a traditional medicine [1]. All parts of this tree are traditionally used, and the fruit in particular has been known to nourish the liver and kidneys and has been used for the treatment of eye and skin disorders, such as tinnitus and blurred vision. It is also used for the prevention of gray hair. Previous phytochemical studies showed that its main constituents are secoiridoids, triterpene and phenolic derivatives. It is reported to have anti-inflammatory, anticancer, antibacterial, and antioxidant activities [2,3,4,5].

Inflammation is an innate immune response to maintain normal conditions triggered by a variety of factors, including pathogens, damaged cells and cytokines [6,7]. However, uncontrolled inflammation is a cause of various diseases, such as arthritis, asthma, and various skin diseases [8,9]. Macrophages stimulated by lipopolysaccharide (LPS) secrete inflammatory mediators such as nitric oxide (NO) and prostaglandin E2 (PGE2) together with cytokines such as tumor necrosis factor (TNF-α) and interleukins (ILs) [10,11]. The cellular expression of pro-inflammatory cytokines is regulated by many factors, and Nuclear factor kappa B (NF-κB) is considered one of the key transcriptional factors in cytokine expression. Mitogen-activated protein kinases (MAPKs) are also regarded as important for the regulation of many transcriptional factors, including NF-κB [12,13,14]. Therefore, inhibitors that suppress pro-inflammatory cytokines are considered as inflammatory therapeutics. In addition, NF-κB and MAPKs are important targets for developing anti-inflammatory materials [15,16].

Inflammation of the skin occurs due to various causes, including infections or allergens. The inflammatory response plays a crucial role in the development of chronic skin disease, including atopic dermatitis, psoriasis, and infectious diseases, which are accompanied by itching and swelling. Psoriasis is a chronic skin disease that causes thick stratum corneum due to abnormal epidermis cell differentiation. Psoriasis accompanies a high expression level of inflammatory cytokines, which cause pathological symptoms [17,18]. TNF-α initiates the secretion of IL-1α/β, IL-6 and chemokine ligand 20 (CCL20) through NF-κB and the signal transducer and activator of transcription 3 (STAT3) pathways. IL-17A secreted by Th17 cells increases the expression of keratin 17, causing skin hyperkeratosis. CCL20 stimulates an immune response in skin by attracting immune cells [19,20,21]. 

The fruits *of L. ludicum* have been used for the regulation of inflammation and skin-related disorders in traditional medicines. However, active constituents to support the beneficial therapeutic potential for inflammation-related skin diseases are not well-characterized. Therefore, an investigation was conducted to identify the anti-inflammatory constituents of *L. ludicum* fruits. In addition, the anti-inflammatory activity of the isolated compounds was assessed by evaluating the NO production of LPS-stimulated RAW264.7 macrophages. The mRNA expression of cytokines and effects on NF-κB and MAPK signaling pathway were also measured. Moreover, the anti-inflammatory activity of active constituents on a TNF-α/IL-17A/IFN-γ-stimulated human keratinocyte psoriasis model was also evaluated. 

## 2. Results

### 2.1. Isolation of Compounds from the Fruits of L. lucidum

**Figure 1:**
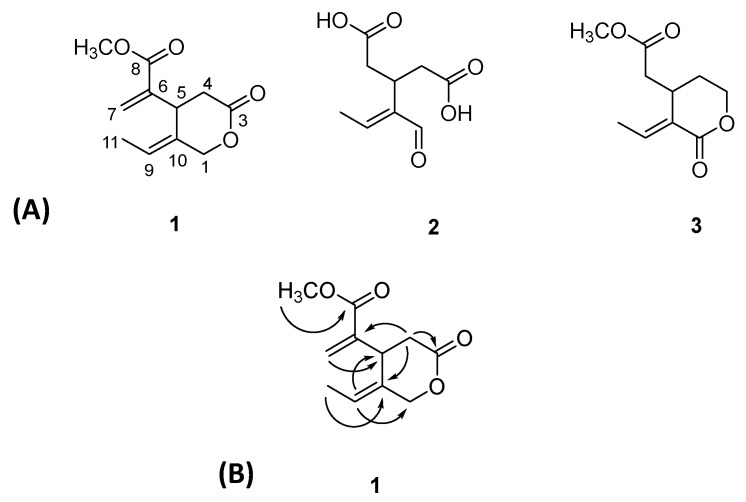
(**A**) Chemical structures of compounds **1**–**3** of *L. lucidum* fruits and (**B**) Key HMBC (→) correlations of new compound **1.**

Three compounds were isolated from the *n*-BuOH fraction of *L. lucidum* fruits by chromatographic separation (Figure 1). The structures of isolated compounds were elucidated by an extensive spectroscopic data analysis together with comparison with literature values.

#### 2.1.1. Structural Determination of New Compound

Compound **1** was purified as an amorphous gum. The molecular formula was deduced as C_11_H_14_O_4_ from the HRESI-MS (*m*/*z* 233.0784 [M + Na]^+^, calcd for C_11_H_14_NaO_4_^+^, 233.0784), which was verified by its ^13^C NMR data. The ^1^H NMR spectrum, together with its corresponding carbons in the HSQC spectrum, suggested the presence of an oxymethylene at [δ_H_ 4.78 (1H, d, *J* = 9.8 Hz, H-1a) and 4.74 (1H, d, *J* = 9.8 Hz, H-1b); δ_C_ 72.3], a methylene at [δ_H_ 2.91 (1H, dd, *J* = 15.4, 6.3 Hz, H-4a) and 2.75 (1H, dd, *J* = 15.4, 6.3 Hz, H-4b); δ_C_ 35.2], two methines at [δ_H_ 4.01 (1H, t, *J* = 6.3 Hz, H-5); δ_C_ 35.8] and [δ_H_ 5.82 (1H, q, *J* = 5.6 Hz, H-9); δ_C_ 125.6], an *exo*-methylene at [δ_H_ 6.31 (1H, s, H-7a), δ_H_ 5.75 (1H, s, H-7b); δ_C_ 125.9], a methyl group at [δ_H_ 1.56 (1H, d, *J* = 5.6 Hz, H-11); δ_C_ 125.6] and a methoxy group at [δ_H_ 3.80 (3H, s); δ_C_ 51.2] (Table 1). Additionally, two additional carbonyl carbons and two quaternary carbons were observed at [δ_C_ 173.7 and 166.4] and [δ_C_ 140.6 and 131.9] in the ^13^C NMR spectrum, respectively. Based on these data, compound **1** was supposed to be an secoiridoid, which has a structure similar to *trans*-(*E*)-3-(1-oxobut-2-en-2-yl)glutaric acid (**3**) [22]. However, compound **1** possessed *exo*-methylene signals instead of a methylene group in compound **3**. The position of the *exo*-methylene group was determined by the HMBC correlations of H-7/C-5 and H-4/C-6. In addition, HMBC correlations of H-4/C-3 determined the position of carbonyl grout at C-3. Taken together, compound **1** was determined to be a new secoiridoid, as shown in Figure 1, and named secoligulene (**1**).

#### 2.1.2. Identification of Known Compounds

Two known compounds were identified as (*E*)-3-(1-oxobut-2-en-2-yl)pentanedioic acid (**2**) and *trans*-(*E*)-3-(1-oxobut-2-en-2-yl)glutaric acid (**3**) based on the spectroscopic analysis and comparison with previous values [22,23].

### 2.2. Anti-Inflammatory Effects on LPS-Stimulated RAW264.7 Cells 

#### 2.2.1. Inhibitory Effect of Compounds **1**–**3** on the Production of NO and Cytokines

**Figure 2:**
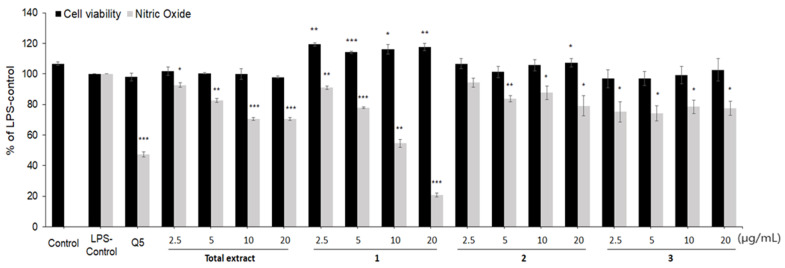
Effects of *L. lucidum* fruits extract and compounds **1–3** on the NO production and cell viability in LPS-stimulated RAW264.7 macrophages. All data are presented as the mean ± SEM (*n* = 3). Quercetin (Q5, 5 μg/mL) was used as the positive control. * *p <* 0.05, ** *p <* 0.01 and *** *p <* 0.001 indicate significant differences compared to the LPS control group.

During the inflammatory process, diverse inflammatory mediators such as NO, PGE2 and cytokines were secreted by the macrophages, which triggers additional detrimental responses. Therefore, the anti-inflammatory activity of the total extract of *L. lucidum* fruits and its isolated compounds (**1**–**3**) was first evaluated by measuring NO production using Griess assay in LPS-stimulated RAW 264.7 macrophages. Before the evaluation of anti-inflammatory activity, a cytotoxicity experiment was performed to confirm the nontoxic concentration of the samples. As shown in Figure 2, the total extract of *L. lucidum* and three isolated compounds did not show any cytotoxicity up to 20.0 μg/mL. The treatment of RAW 264.7 macrophages with LPS increased the production of NO, which was dose-dependently reduced by the treatment of total extract of *L. lucidum* fruits and compounds **1**–**3**. Among the three compounds, a new compound, secoligulene (**1**), showed the most potent inhibition on NO production with an IC_50_ value of 12.0 μg/mL.

**Figure 3:**
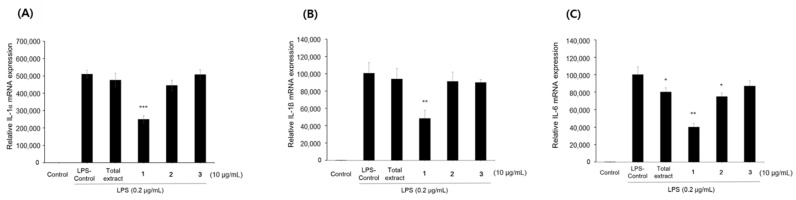
Effects of *L. lucidum* fruits extract and compounds **1**–**3** on the mRNA expression of cytokines, (**A**) IL-1α, (**B**) IL-1β and (**C**) IL-6, in LPS-stimulated RAW264.7 macrophages. All data are presented as the mean ± SEM (*n* = 3). Quercetin (Q5, 5 μg/mL) was used as the positive control. * *p <* 0.05, ** *p <* 0.01 and *** *p <* 0.001 indicate significant differences compared to the LPS control group.

To verify the anti-inflammatory effect of total extract of *L. lucidum* fruits and compounds **1**–**3**, their effects on the production of cytokines were also evaluated. The mRNA expression of inflammatory cytokines genes such as IL-1α, IL-1β and IL-6 was significantly increased in LPS-stimulated RAW264.7 cells compared to control. Consistent with the results of NO assay, compound **1** significantly inhibited the mRNA expression of inflammatory cytokines, including IL-1α, IL-1β and IL-6 (Figure 3). However, the total extract and compounds **2**–**3** showed a weak activity.

#### 2.2.2. Effect of Compound **1** on NF-κB Pathway and MAPK/STAT3 Signaling

**Figure 4:**
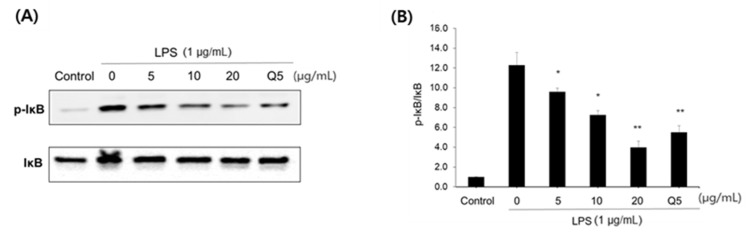
Effect of compound **1** on the IĸB phosphorylation in the NF-κB signaling pathway. (**A**) Western blots of p-IĸB and IĸB and (**B**) quantitative expression of p-IĸB/IĸB. All data are presented as the mean ± SEM (*n* = 3). Quercetin (Q5, 5 μg/mL) was used as the positive control. * *p <* 0.05 and ** *p <* 0.01 indicate significant differences compared to the LPS control group.

Among the three isolated compounds, compound **1** showed the most potent inhibitory activity. Therefore, further experiments were performed to investigate the anti-inflammatory potential of compound **1**. As the NF-κB/I-κB complex is considered to be one of the major transcription regulators of inflammatory cytokine expression, its effect on the NF-κB pathway was evaluated using LPS-induced RAW 264.7 macrophages. Phosphorylated I-κB was increased by treatment with LPS in comparison with the untreated group, while the protein expression of I-κB was not changed. However, treatment with compound **1** dose-dependently reduced the expression of p-IκB (Figure 4A,B). 

**Figure 5:**
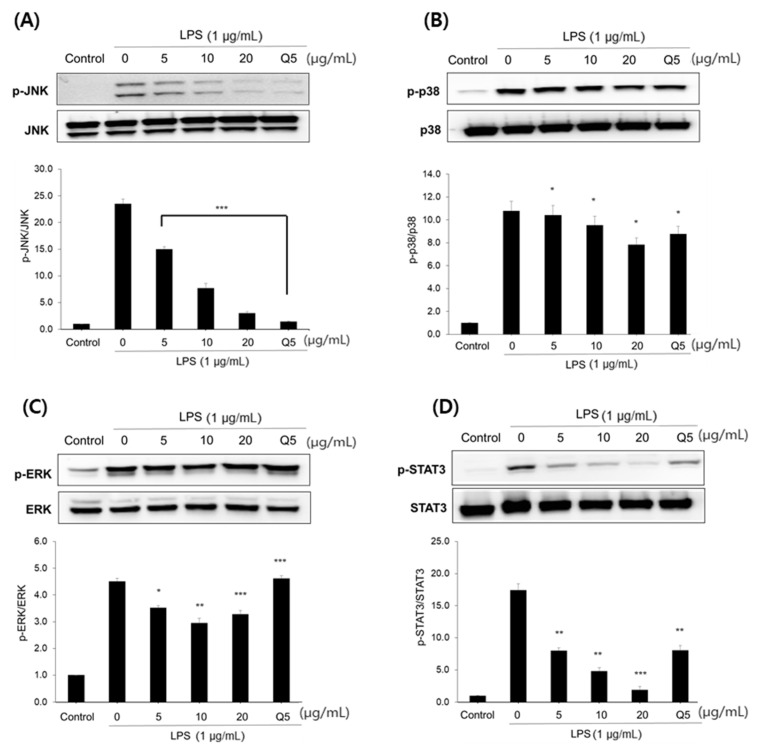
Effect of compound **1** on the phosphorylation of MAPK, (**A**) JNK, (**B**) p38, (**C**) ERK and (**D**) STAT3 in RAW 264.7 cells. All data are presented as the mean± SEM (*n* = 3). Quercetin (Q5, 5 μg/mL) was used as the positive control. * *p* < 0.05 and ** *p* < 0.01, *** *p* < 0.001 indicate significant differences compared to the LPS control group.

LPS also activates the MAPK downstream cell signaling pathway by stimulating Toll-like receptor 4 (TLR4) on the surface of macrophages [12,13]. The activated signaling pathway induces the expression of inflammatory mediators, such as pro-inflammatory cytokines, NO and many transcriptional factors, including NF-κB [14]. Therefore, we tested whether compound **1** exerted an anti-inflammatory effect through the MAPKs pathway. The increased phosphorylation of ERK, p38 and JNK by LPS was significantly inhibited by the treatment with compound **1** in a dose-dependent manner (Figure 5A–C). In particular, JNK phosphorylation was most effectively inhibited among the three MAPKs. STAT3 is another important factor involved in the inflammatory responses. Compound **1** also significantly suppressed STAT3 phosphorylation in LPS-stimulated RAW264.7 cells (Figure 5D). Taken together, the anti-inflammatory effect of compound **1** was achieved in part by the inhibition of MAPK and STAT3 phosphorylation.

### 2.3. Effects on Psoriasis Regulation in TNF-α/IL-17A/IFN-γ Induced HaCaT Cells

**Figure 6:**
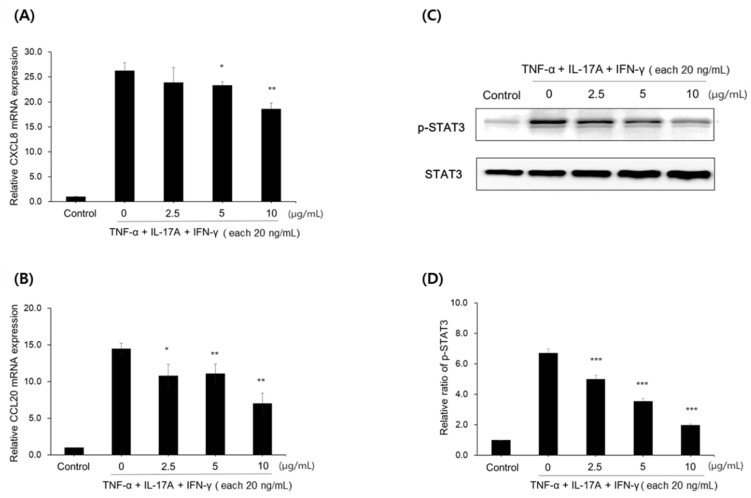
Effect of compound **1** on chemokines and STAT phosphorylation in TNF-α /IL-17A/ IFN-γ induced HaCaT cells. (**A**) CXCL8, (**B**) CCL20 mRNM expression and (**C**) western blots of p-STAT3 and STAT3 and (**D**) quantitative expression of p-STAT3/STAT3. All data are presented as the mean ± SEM (*n* = 3). * *p* < 0.05 and ** *p* < 0.01, *** *p* < 0.001 indicate significant differences compared to the TNF-α /IL-17A/ IFN-γ control group.

Psoriasis is a chronic inflammatory skin disease and expresses high levels of inflammatory cytokines. Therefore, we further evaluated the effect of compounds **1**–**3** in a psoriasis model. TNF-α /IL-17A/ IFN-γ are known to be psoriasis-inducing mediators in the immune system of skin. TNF-α triggers the secretion of inflammatory cytokines (IL-1α/β, IL-6, IL-23) and CCL20 through STAT3 and NF-κB pathways. IL-17A is one of cytokines secreted by Th17 cells. IL-17A increases the expression of keratin-17 and IL-6/8 through the STAT 1/3 pathway in keratinocytes. Thus, TNF-α/IL-17A/IFN-γ induced a psoriasis condition, ultimately causing skin hyperkeratosis [24,25]. As shown in Figure 6, the levels of chemokines such as CXCL8 and CCL20 were dramatically increased in the TNF-α/IL-17/IFN-γ-induced HaCaT psoriasis model. However, treatment with compound **1** significantly reduced the production of these chemokines in a dose-dependent manner. Compound **1** also clearly inhibited the phosphorylation of STAT3, which regulates the expression of cytokines, and CCL20 chemokines in TNF-α /IL-17A/ IFN-γ induced the psoriasis condition. These data suggest that compound **1** inhibited the production of chemokines by the regulation of STAT3 phosphorylation in the HaCaT psoriasis model.

### 2.4. Anti-Inflammatory Potential of L. ludicum and Its Secoirioids 

In this study, the anti-inflammatory potential of *L. lucidum* was investigated. Three secoiridoids (**1**–**3**), including one new compound, were isolated from the fruits of *L. ludicum* and anti-inflammatory activity was assessed. 

The anti-inflammatory activity was evaluated by the measurement of the production of NO and inflammatory cytokines in LPS-stimulated RAW264.7 cells. NO has long been considered as a key mediator of various inflammation and its related diseases. The role of NO in skin diseases has also been reported. NO production was increased in atopic dermatitis and allergic skin diseases, and further induced inflammatory responses [26,27]. In addition, increased NO production was also observed in the skin of psoriasis patients, which suggests a correlation between the amount of NO and the severity of psoriasis [28]. The administration of NO inhibitors alleviates inflammatory responses; therefore, it has become a therapeutic strategy for skin inflammation [29]. The *L. ludicum* extract significantly inhibited the production of NO in LPS-stimulated RAW 264.7 macrophages. Three compounds isolated from *L. ludicum* fruits also inhibited NO production but showed differences in efficacy. Compound **1** showed the most potent inhibitory activity, whereas the efficacy of compounds **2** and **3** was relatively weak. The anti-inflammatory effect of compound **1** was also confirmed by lowering the production of other inflammatory cytokines IL-1α, IL-1β and IL-6. All the three compounds belong to secoiridoids and share similar skeleton but exerted differential activity. Therefore, the structure of compound **1**, such as presence of *exo*-methylene, is thought to be important, which needed to clarified by further study. 

NF-κB is important transcription factor that is involved in the signal pathway of inflammation responses [30]. MAPK and STAT signaling are also known to be crucial for the production of cytokines in macrophages [13,31]. The JNK pathway is especially important for the release of inflammatory cytokines. Therefore, the effect of secoligulene (**1**), which showed a potent NO inhibitory activity, on the signaling pathway was investigated. Secoligulene (**1**), dose-dependently attenuated the phosphorylation of IκBα (Figure 4). The phosphorylation of MAPK and STAT3 was also significantly reduced by the treatment of secoligulene (**1**). Secoligulene (**1**) strongly suppressed the activation of JNK and STAT (Figure 5A,D). Therefore, compound **1** was suggested to exert an anti-inflammatory effect by the inhibition of MAPK and STAT3 phosphorylation.

Psoriasis is chronic immune skin disease with persistent inflammation. Cytokines and inflammatory factors from immune cells induce inflammatory responses in keratinocytes, which promotes further psoriatic inflammation [32]. Therefore, controlling keratinocyte inflammation using anti-inflammatory therapeutics is suggested to be effective in the management of symptoms [33]. A further investigation of the effect of compounds **1** on psoriasis showed a dramatic decrease in the levels of chemokines, CCL8 and CCL20 in the TNF-α/IL-17/IFN-γ-induced HaCaT psoriasis model. Compound **1** also inhibited the phosphorylation of STAT3, which regulates the expression of cytokines and chemokine in the psoriasis condition (Figure 6). These data suggest that compound **1** inhibited the production of chemokines by the regulation of STAT3 phosphorylation in HaCaT psoriasis model. 

As described above, extracts of *L. ludicum* fruit and its components exhibited anti-inflammatory effects and were effective in a model of psoriasis. The components isolated from the *L. ludicum* fruit are secoiridoid derivatives, which are also abundantly contained in olives and the Oleaceae family [34,35]. Therefore, this study can be used to identify the efficacy of *L. ludicum* fruits and various plants containing secoiridoid derivatives for inflammation-related skin diseases. 

## 3. Materials and Methods

### 3.1. General Experimental Procedure 

The UV and IR spectra A were obtained using Jasco UV-550 and Perkin-Elmer model LE599 spectrometer, respectively. A Bruker DRX 500 or 700 MHz spectrometer were used for the analysis of NMR signals using methanol-*d*_4_ as solvents. ESIMS data were measured on VG Autospec Ultima Mass spectrometers. 

A RAW264.7 mouse macrophage cell line was obtained from Korean Cell Line Bank (No. 40071, Seoul, Korea), and a HaCaT human keratinocyte cell line was obtained from Cell Lines Service GmbH (No. 300493, Eppelheim, Germany). Griess reagent was purchased from Sigma Aldrich Co. (St. Louis, MO, USA). A TaqMan probe was obtained from Applied Biosystems (Foster City, CA, USA). To confirm the change in protein level, anti-IκB-α, anti-phospho-IκB-α, anti-ERK1/2, anti-phospho-ERK1/2, anti-JNK, anti-phospho-JNK and anti-GAPDH were obtained from Invitrogen (Carlsbad, CA, USA) and anti-p38 MAPK, anti-phospho-p38 MAPK, anti-STAT3 and anti-phospho-STAT3 were purchased from Cell Signaling (Danvers, MA, USA).

### 3.2. Plant Material

The fruits of *L. lucidum,* which were cultivated in Jeju Province of Korea, were purchased from a herbal market (Jecheon, Korea). The specimen was identified by the herbarium of the College of Pharmacy, where voucher specimens (CBNU2016-LLF) were deposited. The collection of plant materials received permission from the farmer, and the study complied with the institutional regulations and guidelines.

### 3.3. Extraction and Isolation of Compounds 

The air-dried fruits of *L. ludicum* (5 kg) were extracted with 80% methanol at room temperature and concentrated in vacuo to yield a total extract of 1052 g. The methanol extract was dissolved in water and successively partitioned with *n*-hexane, CH_2_Cl_2_, EtOAc and *n*-BuOH. The *n*-BuOH fraction (227 g) was subjected to Diaion HP-20 column chromatography using gradient mixtures of water–methanol (100:0, 80:20, 60:40, 40:60, 20:80, 0:100) as eluents to give six fractions (LLB1-LLB6). 

LLB2 was subjected to silica gel column chromatography, eluted with gradient mixtures of CH_2_Cl_2_ and MeOH (100:1 to 1:100), to give eight fractions (LLB2A-LLB2H). LLB2B was chromatographed on a Sephadex-LH20 using 100% MeOH to afford eight subfractions (LLB2E1-LLB2E8). Semi-preparative HPLC (Phenomenex Gemini-NX, 5 μm, C18, 10 × 150 mm) of LLB2E1 using MeCN-H_2_O (25:75, flow rate 2 mL/min) yielded compound **2** (12.5 mg). 

LLB4 was subjected to a silica gel column chromatography using CH_2_Cl_2_-MeOH, and the eluate was separated into seven fractions (LLB4A—LLB4G). LLB4B was chromatographed on a Sephadex-LH20 using 100% MeOH to afford sixteen subfractions (LLB4B1-LLB4B16). Compounds **1** (3.1 mg) and **3** (15.2 mg) were purified by semi-preparative HPLC (Phenomenex Gemini-NX, 5 μm, C18, 10 × 150 mm) using MeCN-H_2_O (25:75, flow rate 2 mL/min) from LLB4B3 and LLB4B13, respectively. 

*Secoligulene* (**1**) Amorphous syrup; IR_max_ 3702, 2965, 1727 cm^−^¹; ^1^H NMR (CD_3_OD, 700 MHz) and ^13^C NMR (CD_3_OD, 175 MHz), see Table 1 and Supplementary Materials: Figure S1–S4; ESI-MS (positive node) *m*/*z* 233 [M+Na]^+^; HRESIMS (positive node) *m*/*z* 233.0784 (calcd for C_11_H_14_NaO_4_^+^, 233.0784).

### 3.4. Evaluation of Anti-Inflammatory Activity 

#### 3.4.1. Effect of Compound 1 on MAPK/STAT3 Signaling

A RAW264.7 mouse macrophage cell line and HaCaT human keratinocyte cell line were used for anti-inflammatory studies. Both lines were cultured using an incubator containing 5% CO_2_ at 37 °C, and Dulbecco’s Modified Eagle’s Medium (DMEM) supplemented with 10% fetal bovine serum, 100 U/mL penicillin, and 100 µg/mL streptomycin.

#### 3.4.2. Cell Viability Assay

Cytotoxicity was measured using a Cell Counting Kit-8. Each cell line was dispensed in a 24-well plate at a density of 1 × 10^5^ cells/well, and then incubated for 24 h at 37 °C. Compounds **1**–**3** were diluted in DMEM media for each concentration and incubated for 24 h. After removing the existing medium, a 10:1 diluted solution of phenol red-free DMEM and CCK-8 reagent was dispensed and incubated for 30 min. Measurements were performed at 450 nm using a microplate reader. 

#### 3.4.3. Measurement of NO 

RAW264.7 macrophages were cultured on a 24-well plate at a density of 1 × 10^5^ cells/well and stabilized for 24 h. Cells were treated with different concentrations of compounds **1**–**3** in the presence of LPS for 24 h. Cultured supernatant and Griess reagent were mixed 1:1, and incubated for 10 min at room temperature. Absorbance values at 540 nm were recorded using a microplate reader.

#### 3.4.4. Real-Time RT-PCR 

RAW264.7 cells and HaCaT cells were cultured on 6-well plates at 5 × 10^5^ cells/well. To stimulate inflammatory reaction, RAW264.7 cells and HaCaT cells were treated with LPS for 6 h and TNF-α, IL-17A, IFN-γ for 24 h, respectively. Compounds were simultaneously added in different concentrations as indicated. A total of 1.5 μg of RNA was used to make cDNA using a Revertra ACE kit. Real-time RT-PCR experiments were conducted using a StepOne real-time RCR instrument and a TaqMan probe. The amount of gene mRNA expression amplified in real time was comparatively analyzed using a TaqMan probe for each gene. Expression values were normalized to GAPDH: IL-1α (Mm00439620_m1); IL-1β (Mm00434228_m1); IL-6 (Mm00446190_m1); COX-2 (Mm00478374_m1); GAPDH (Mm99999915_g1); IL-1α (Hs00174092_m1); IL-1β (Hs01555410_m1); IL-6 (Hs00174131_m1); TNF-α (Hs00174128_m1); CXCL8 (Hs00174103_m1); CCL20 (Hs01011368_m1); GAPDH (Hs02786624_g1).

#### 3.4.5. Western Blot Analysis

For protein extraction, the cells were lysed and eluted using RIPA buffer (25 mM Tris•HCl pH 7.6, 150 nM NaCl, 1% NP-40, 1% sodiumdeoxycholate, 0.1% SDS phosphatase inhibitor cocktail, and 50 mM EDTA) (Thermo Fisher Scientific, Waltham, MA, USA). After quantifying using Bradford assay, it was heated with laemmli sample buffer (31.5 mM Tris-HCl, pH 6.8, 10% glycerol, 1% SDS, 0.005% Bromophenol Blue) and β-mercaptoethanol (Sigma Aldrich Co., St. Louis, MO, USA). at 95 ℃ for 5 min. Then, the protein samples were separated in 10% SDS PAGE and transferred to PVDF membrane. The membrane was blocked with 10% skim milk prepared in TBST and then incubated for 1 h at room temperature. Protein phosphorylation was detected using specific primary antibodies: anti-IκB-α (Invitrogen, 39-7700); anti-phospho-IκB-α (Invitrogen, MA5-15087); anti-p38 MAPK (CST, 9212); anti-phospho-p38 MAPK (CST, 9211); anti-ERK1/2 (Invitrogen, 13-6200); anti-phospho-ERK1/2 (Invitrogen, 700012); anti-JNK (Invitrogen, MA5-15183); anti-phospho-JNK (Invitrogen, MA5-15228); anti-STAT3 (Cell Signaling Technology, 4904); anti-phospho-STAT3 (Cell Signaling Technology, 9145); anti-GAPDH (Invitrogen, MA5-15738). Next, the antibodies were washed in TBST, incubated with a secondary antibody (1:10,000; Invitrogen) for 1 h at room temperature, and treated with ECL solution. The images were observed with an image processing device and analyzed with processing using the Image J program (See Supplementary Materials: Figure S5–S7).

### 3.5. Statistical Analysis

All data are presented as the mean ± SEM (*n* = 3). Significant differences were analyzed by one-way ANOVA or Student’s t-test where appropriate. As a result, *p < 0.05 was considered statistically significant.

## 4. Conclusions

*L. ludicum* has been used for the regulation of inflammation and skin-related disorders. Therefore, we tried to find active compounds to support traditional usage. In this study, three secoiridoids (**1**–**3**), including one new compound, were isolated, and their structures were elucidated based on spectroscopic analysis. Further biological assessment revealed the anti-inflammatory activity of the isolated compounds, as measured by the production of NO and inflammatory cytokines in LPS-stimulated RAW264.7 cells. In particular, a new compound, secoliguene (**1**), showed the most potent anti-inflammatory activity. The investigation of the signaling pathway suggested that compound **1** dramatically inhibited the phosphorylation of I-κB, MAPK and STAT3 in RAW264.7 cells stimulated by LPS. In addition, compound **1** also effectively reduced the chemokines and STAT3 phosphorylation in psoriasis HaCaT cells. Taken together, these findings support the beneficial effects of *L. lucidum* and its constituents on inflammation-related skin diseases and can be further be developed as therapeutic treatment for the related diseases. 

## Figures and Tables

**Table 1:** NMR spectroscopic data for compound **1** (methanol-*d_4_*).

Carbon Number	δ_H_ (700 MHz)	δ_C_ (175 MHz)
1	4.78 (1H, d, *J* = 9.8 Hz)	72.3
	4.74 (1H, d, *J* = 9.8 Hz)	
3	-	173.7
4	2.91 (1H, dd, *J* = 15.4, 6.3 Hz)	35.2
	2.75 (1H, dd, *J* = 15.4, 6.3 Hz)	
5	4.01 (1H, t, *J* = 6.3 Hz)	35.8
6	-	140.6
7	6.31 (1H, s)	125.9
	5.75 (1H, s)	
8	-	166.4
9	5.82 (1H, q, *J* = 5.6 Hz)	125.6
10	-	131.9
11	1.56 (3H, d, *J* = 5.6 Hz)	12.8
OCH	3.80 (3H, s)	51.2

## Data Availability

Data are contained within the article and supplementary materials.

## Supplementary Materials

The following are available online at https://www.mdpi.com/article/10.3390/ph15080932/s1, Figures S1–S4: ^1^H, ^13^C, HSQC, HMBC and HRESI-MS spectrums of new compound **1**, Figures S5–S7: Western blots for isolated compounds.
