# Supplementary material for: Therapeutic Potentials of Secoiridoids from the Fruits of Ligustrum lucidum Aiton against Inflammation-Related Skin Diseases
Source: Pharmaceuticals (Basel). 2022 Jul 27;15(8):932. doi: 10.3390/ph15080932 (PMC9415915; doi:10.3390/ph15080932)
Supplement: Supplementary file 1 [file pharmaceuticals-15-00932-s001.zip › pharmaceuticals-1780020-supplementary.pdf]

Therapeutic Potentials of Secoiridoids from the Fruits of *Ligustrum lucidum* Aiton against Inflammation-Related Skin Diseases

Sang Won Yeon [1,†], Su Ryeon Choi [2,†], Qing Liu [3,†], Yang Hee Jo [1], Da Hee Choi [2], Mi Ran Kim [2], Se Hwan Ryu [1], Solip Lee [1], Bang Yeon Hwang [1], Hyung Seo Hwang [2,*] and Mi Kyeong Lee [1,*]

1 College of Pharmacy, Chungbuk National University, Cheongju 28160, Korea; sangwon1311@naver.com (S.W.Y.); qow0125@naver.com (Y.H.J.); alfm0188@naver.com (S.H.R.); dudaos000@hanmail.net (S.L.); byhwang@chungbuk.ac.kr (B.Y.H.)

2 School of Cosmetic Science and Beauty Biotechnology, Semyung University, Jecheon 27136, Korea; tryu0925@naver.com (S.R.C.); chlekgml930@gmail.com (D.H.C.); alfks4050@naver.com (M.R.K.)

3 Food and Pharmacy College, Xuchang University, Xuchang 461000, China; liuqing7115@hotmail.com

* Correspondence: hshwang@semyung.ac.kr (H.S.H.); mklee@chungbuk.ac.kr (M.K.L.)

† These authors contributed equally to this work.

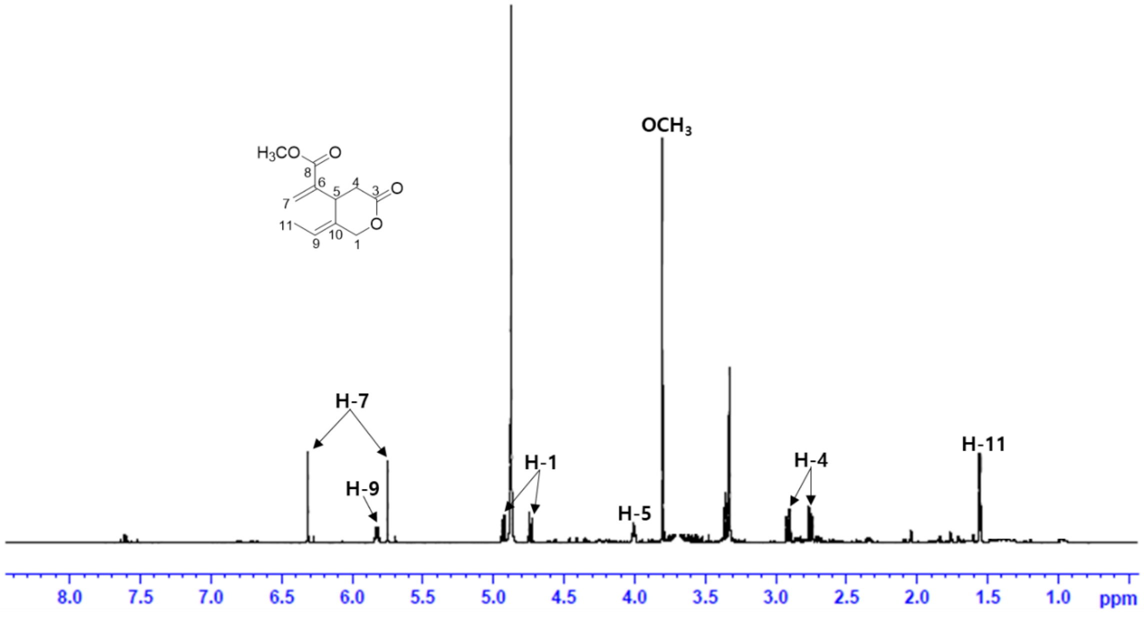

Figure S1. ^{1}H NMR spectrum of compound **1**.

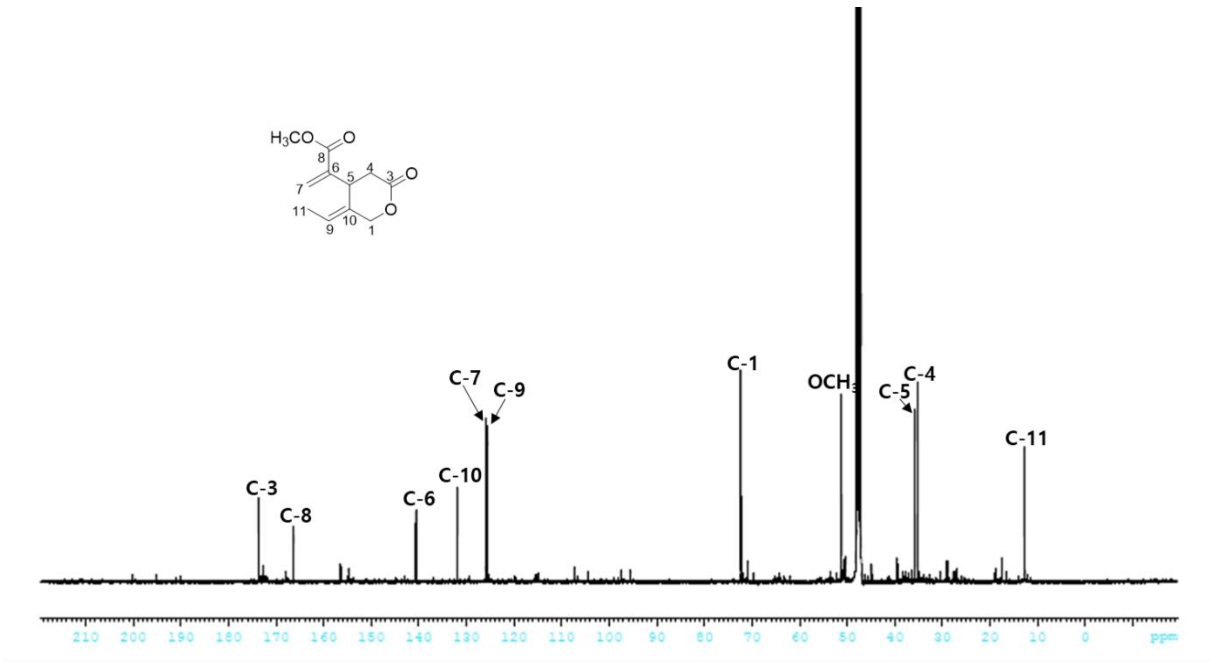

Figure S2. ^{13}C NMR spectrum of compound **1**.

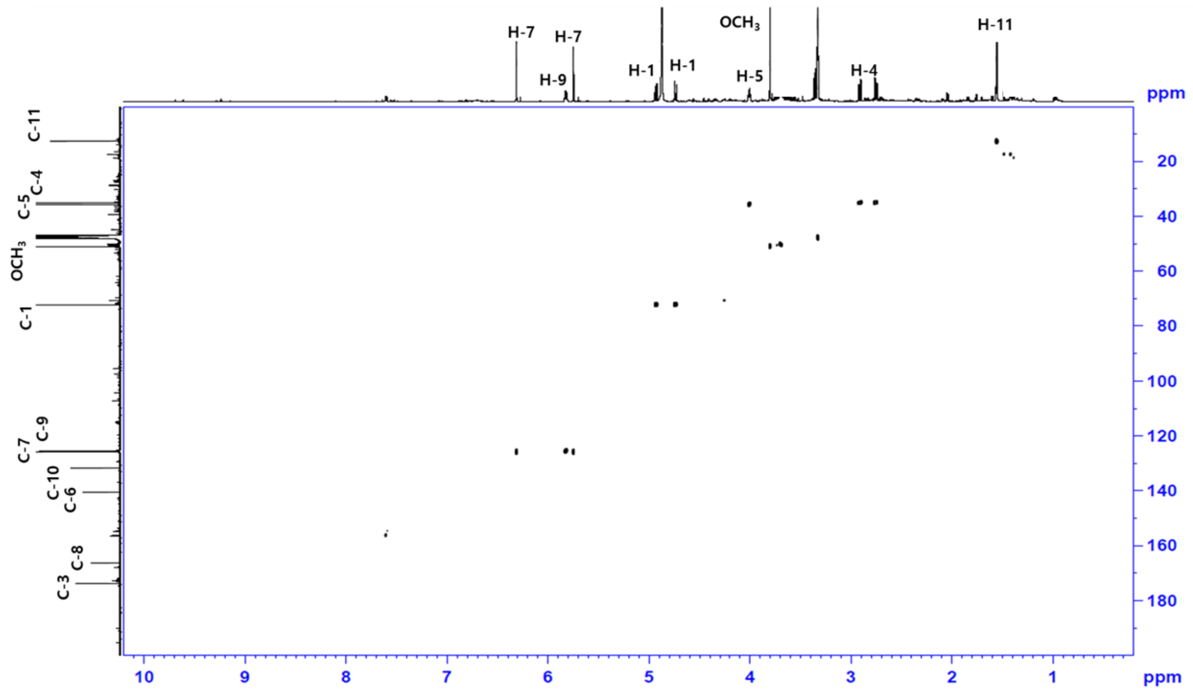

Figure S3. HSQC spectrum of compound **1**.

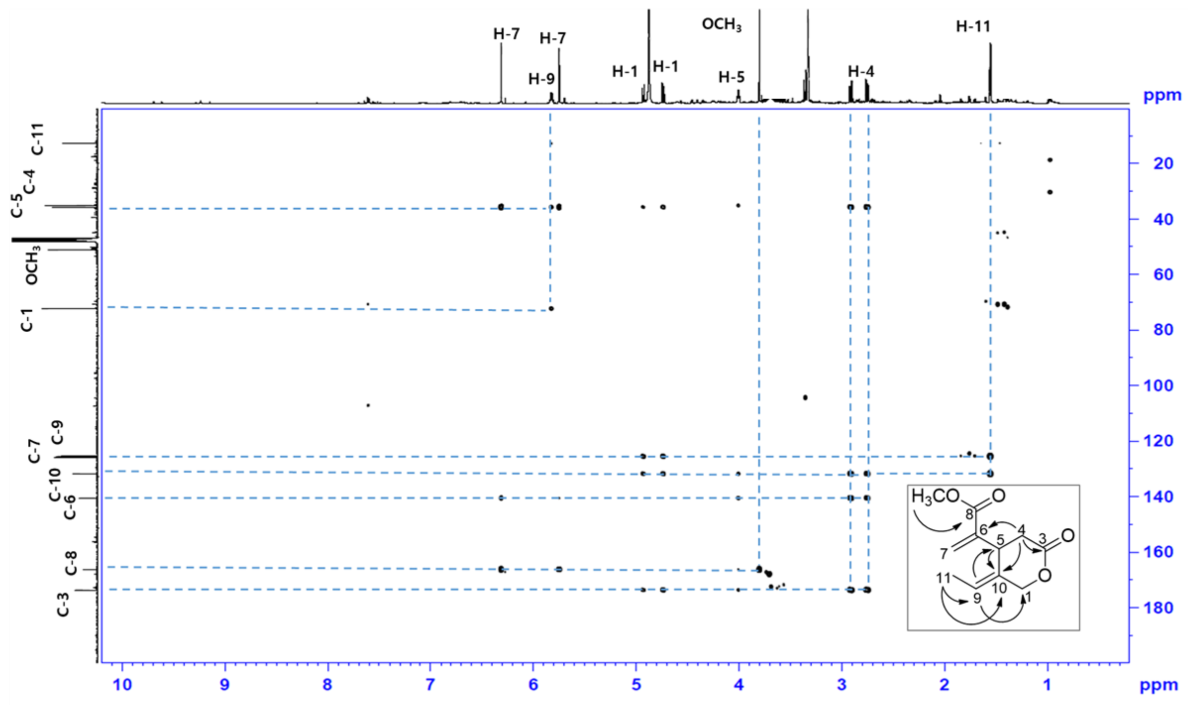

Figure S4. HMBC spectrum of compound **1**.

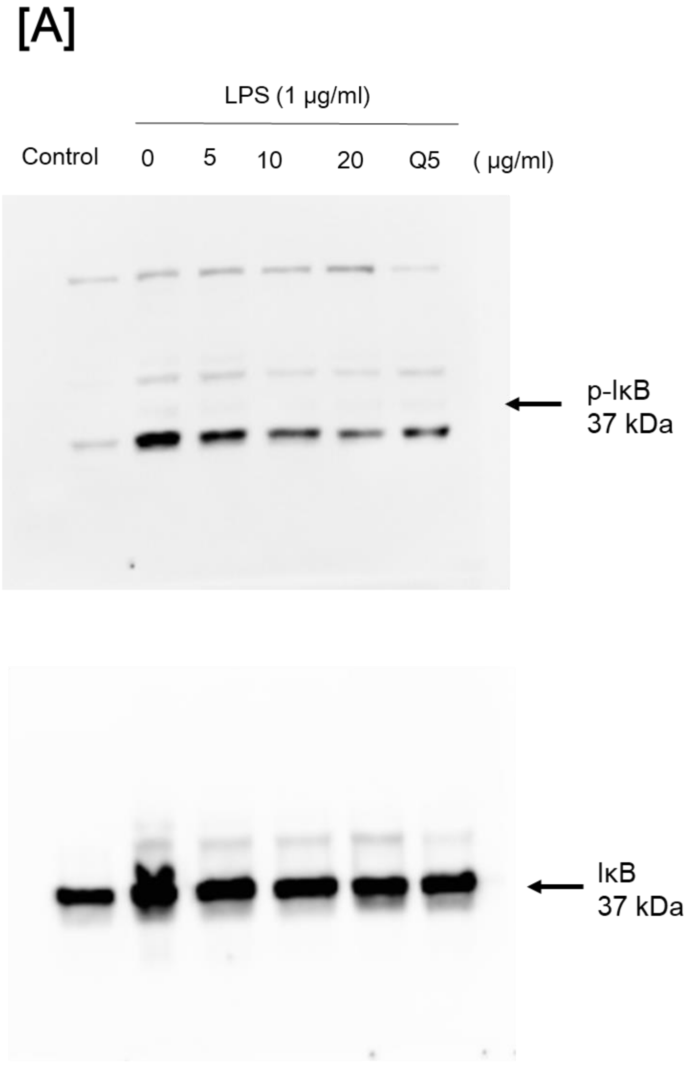

Figure S5. Western blots for the IκB phosphorylation in the NF-κB signaling pathway in LPS induced RAW 264.7 cells (Figure 4 in the manuscript).

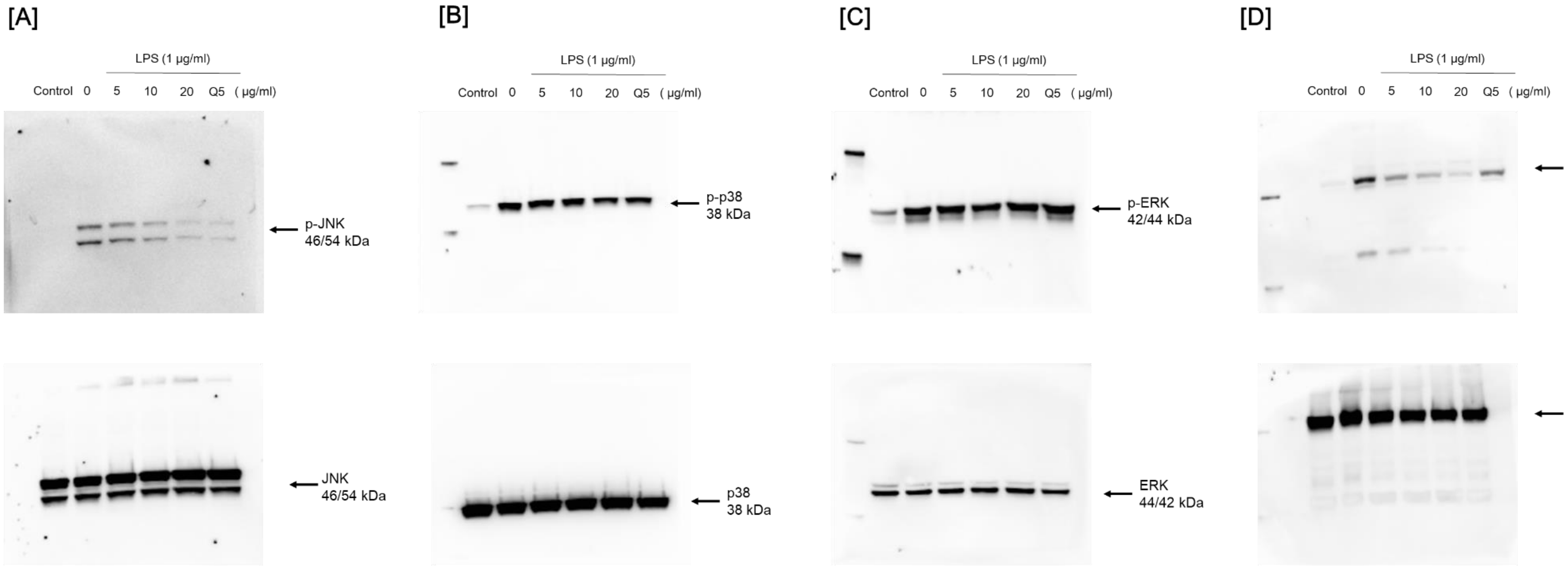

Figure S6. Western blots for the phosphorylation of MAPK and STAT3 in LPS induced RAW 264.7 cells (Figure 5 in the manuscript).

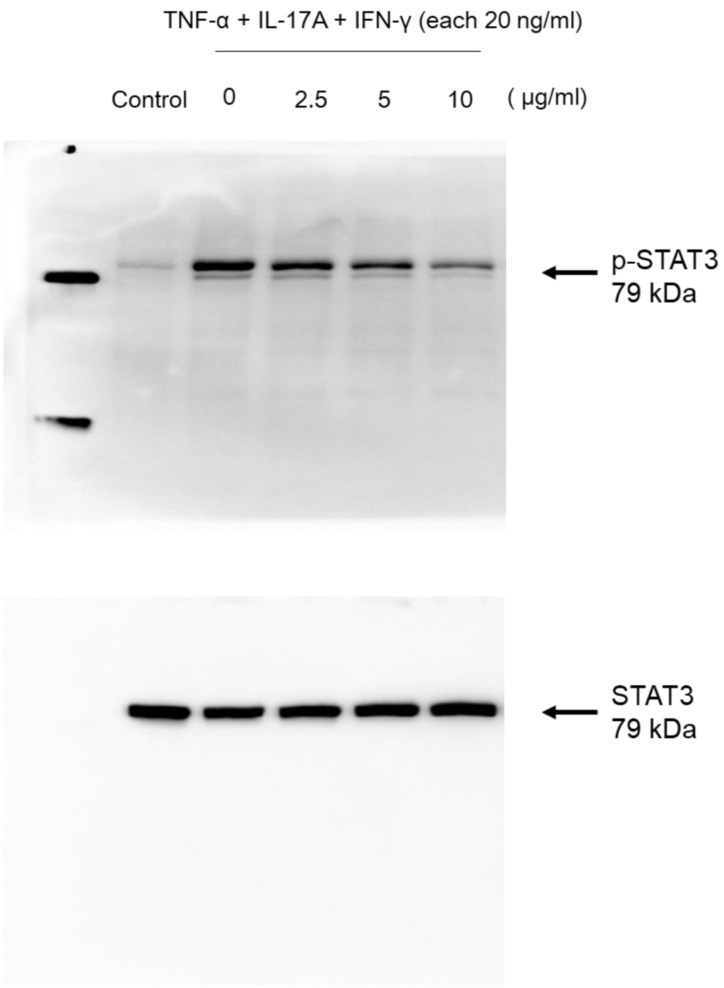

Figure S7. Western blots for the IκB phosphorylation in the NF-κB signaling pathway in TNF-α /IL-17A/ IFN-γ induced HaCaT cells. (Figure 6 in the manuscript).
